# The revolution of metaverse in surgery: a mini-review with video

**DOI:** 10.1007/s13304-024-01960-x

**Published:** 2024-08-05

**Authors:** Michele Ammendola, Riccardo Memeo, Mohanad Al Ansari

**Affiliations:** 1 Science of Health Department, University “Magna Graecia” Medical School, Digestive Surgery Unit, “Renato Dulbecco” Hospital, Viale Europa – Germaneto, 88100 Catanzaro, Italy; 2 Hepato-Biliary and Pancreatic Surgical Unit, “F. Miulli” Hospital, Acquaviva delle Fonti, Bari, Italy; 3 Minimal Invasive Gastrointestinal, Robotic Surgery Unit, Dean of the Robotic Surgery Academy, Aster Hospital, Dubai, UAE; 4 Robotic Surgery Center, Al Zaitoun Specialized Hospital, Baghdad, Iraq

**Keywords:** Metaverse, Virtual reality, Telementoring, Surgery

## Abstract

The virtual reality (VR) is an application in which people can interact each other with their own avatars. Metaverse has already been tested in numerous medical fields and health care as telemedicine, second opinion and remote discussion, but in surgery some fundamental concepts are not yet very widespread. In this study, we want to show our surgery and workshop experiences in the Metaverse to demonstrate the safety and efficiency of this new technology in surgery, in particular for telementoring and remote surgery, combining artificial intelligence (AI), augmented reality (AR) and VR.

**Supplementary Information:**

The online version contains supplementary material available at 10.1007/s13304-024-01960-x.

## Introduction

The term “Metaverse” first appeared in Neal Stephenson’s novel “Snow Crash” in 1992, representing a virtual reality (VR) that goes beyond physical reality. It is a combination of the words “meta” and “universe”, meaning transcendence and virtuality, respectively [1].

The Metaverse appears as a digital world in which people can move through their own reproductions (avatars or digital twins) and interact in real time with other digital people through glasses and dedicated audio–visual devices. Moreover, in this three-dimensional (3D) reality created by the transposition and integration of the real world into the virtual one, people can carry out their actions expressing their own characteristics.

The application of the Metaverse in medicine dates to 2018, when a Medical IoT (MIoT) model was used for lung cancer screening campaign in China. The system compared in real time the tomographic image of the subcentimeter nodules identified in a patient with the previous images present in the archive using a network of special computers. This application, through the five-step assessment of pulmonary nodules, has improved the early diagnosis of pulmonary nodules using big data-based management technologies [2].

Even in surgical field, the concept of Metaverse is gaining ground thanks to the possibility of guaranteeing surgeons easier, more precise, and therefore safer operations.

All these digital data must be processed from a single location that facilitates viewing; this task seems to be already done by the robotic console.

The first step may be to use the Metaverse as a training place. Indeed, the surgical Metaverse is also used to describe a virtual training place that includes all the activities related to surgical procedures. But far beyond that, performing full surgical procedures in VR can be used to train full surgical teams in a secure environment, taking them from novice teams to expert teams. VR allows to create simulations of clinical and diagnostic activities with discussion sessions and research programs by sharing cases with students from all over the world. Physical distances can be filled. This special platform can be used in the development of medical students thanks to the possibility of having virtual models of real organs on which to study, evaluate their relationships with other anatomical structures, and feel their consistency even during sectioning. The training of young surgeons can benefit from the possibility of performing surgery simulations and being guided in their movements remotely.

In this study, we want to demonstrate how VR in surgery is effective and efficient. Effective because it gives advantages within a group such as verbal or pratical telementoring, sharing of peri-operative planning, consultations, clinical and scientific data; efficient, because it gives advantages outside the group, such as improvement of patient outcome, reducing costs and resources for the hospital.

## Materials and methods

**Fig. 1:**
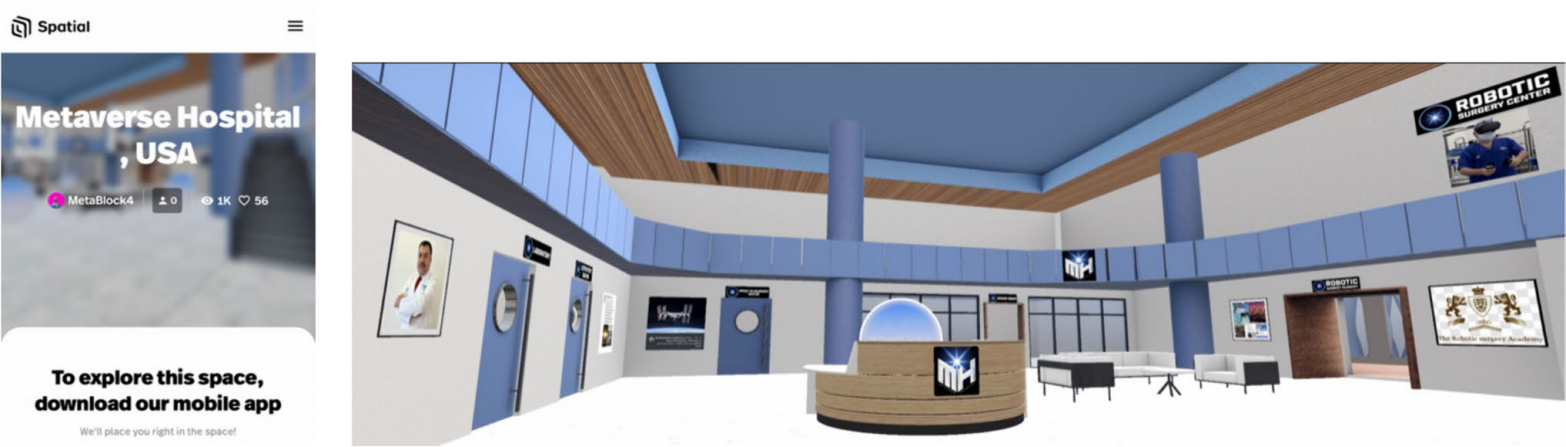
**A** Application and profile. **B** Metaverse Surgical Hospital, “USA”

**Fig. 2:**
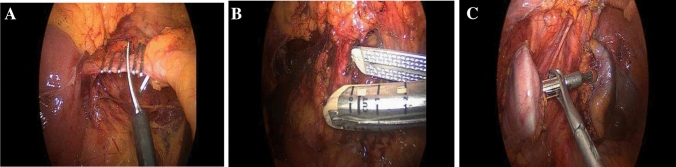
Surgical critical view of safety. **A** Ligation and section of inferior mesenteric vein. **B** Artery. **C** Trans-anal anastomosis

On January 7, 2023, the first Metaverse Surgical Hospital with USA license was inaugurated by Dr. Mohanad Al Ansari (Figs. 1, 2).

Entering in the lobby of the Metaverse Surgical Hospital on the ground floor there are the laboratory, the outpatient clinic, the Space Telesurgery Centre, the board room, the Robotic Surgery Academy room, and the pharmacy; on the second floor there are the Robotic Surgery Centre, the Minimal Invasive Surgery room, and the inpatients rooms. The Metaverse allows the integration between the real hospital and the virtual one, the patients who are admitted and treated in the real hospital can enjoy the care of a global multidisciplinary team and can undergo surgical interventions guided and operated by the best surgeons in the world. The avatar surgeon of Mohanad al Ansari, made telementoring from the Metaverse Hospital operating theater which is connected with the real operation theater in give the name of the hospital. Telementoring is done by Visio-Audio and the system have the ability of AI programs for pre, intra and post-operative phases. The avatar surgeon can use robotic arms controlled from distance by VR of Metaverse Hospital and the arms are in the real Hospital.

On March 2023, during the scientific collaboration between the Translational Research in Surgery Group of “Magna Graecia” University, Italy, and the first Metaverse Surgical Hospital, USA, Ammendola et al. introduced the concept about the development of the Metaverse and its use in the medical field such as VR surgery and telementoring by performing a laparoscopic cholecystectomy and a laparoscopic left colectomy in the virtual hospital from the operative room of the real “Renato Dulbecco” Hospital, Digestive Surgery Unit, Catanzaro, Italy. In constant demonstrative connection, a continuous dialogue between two expert surgeons, was possible to assess each stage of the surgery evaluating the critical view of safety such as Calot triangle, bile duct and artery for cholecystectomy, and mesenteric inferior artery and vein or anastomosis techniques for left colectomy (Videos 1–2) (Fig. 2A–C) [3].

The good results in terms of ease in discussing the surgical steps and participation in the theatre even remotely generated by these first two surgery led us to carry out this second study with the aim of amplifying the concept of low-cost telementoring as an application to real surgery in the Metaverse VR, a patient was selected and underwent robotic hepatectomy during the first HPB Surgery Workshop at Metaverse Surgical Hospital.

On June 28, 2023, the first HPB Surgery Workshop was held at the Metaverse Surgical Hospital, USA. Surgeons and trainees, after downloading the SPATIAL application for virtual reality onto their computers, created their own avatar with a faithful reproduction of their person and had the opportunity to enter the Metaverse Surgical Hospital, USA. There were surgeons from various parts of the world who were able to meet the avatar of Dr Mohanad Al Ansari and all the other participants by setting the audio to converse with each other in high quality voice conversations.

**Fig. 3:**
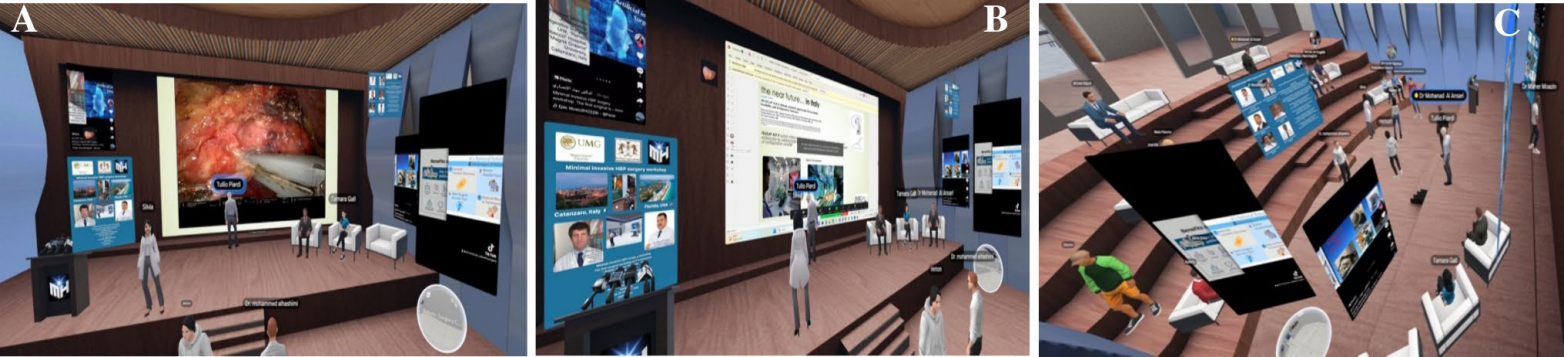
**A** Scientific presentation with video. **B** Scientific oral presentation. **C** Discussion with participants

During the workshop, scientific presentations and various training sessions were held with expert speakers who had HPB surgery as a leitmotiv: diagnosis and treatment of liver, pancreas and biliary tract pathologies between past and present, through robotic surgery up to VR and AI (Video 3) (Fig. 3A, B).

**Fig. 4:**
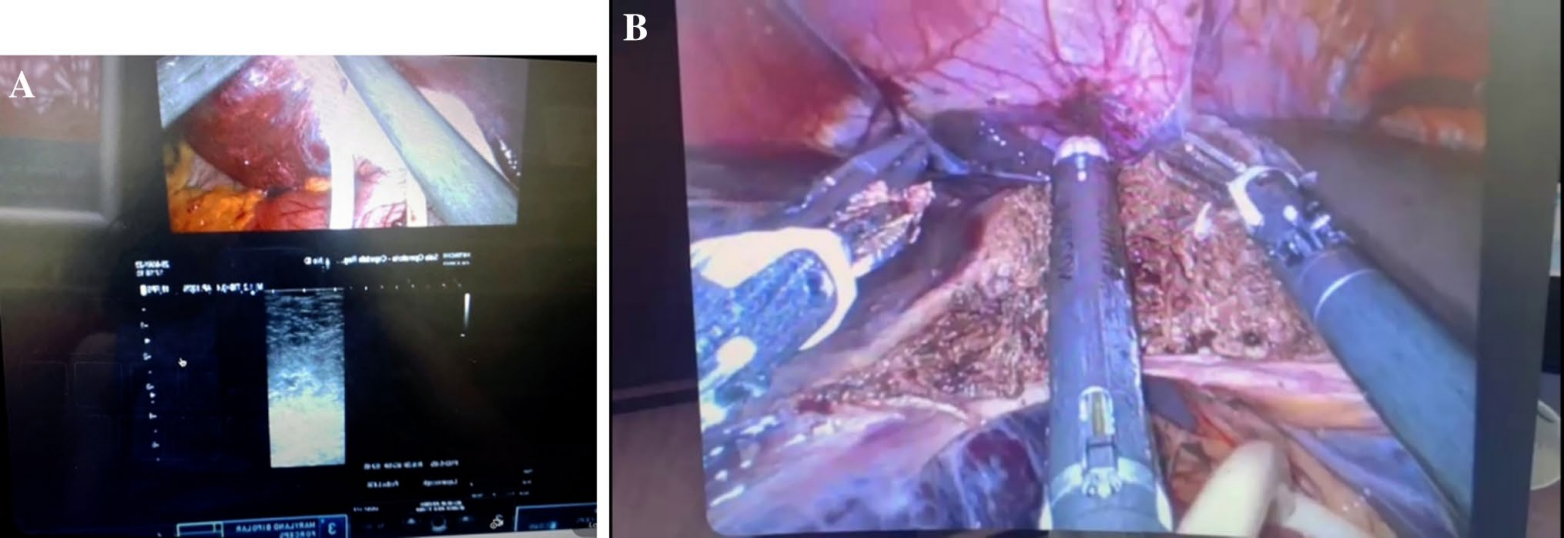
**A** Liver visualization with ultrasound robotic probe. **B** Final liver dissection

Furthermore, a live robotic liver resection was performed at the “Miulli” Hospital of Acquaviva delle Fonti (BA) by Professor Riccardo Memeo (Fig. 4A, B) [4].

## Results

The surgery was performed without distractions and safely. It was easy for the surgeons to intervene during the sessions both by voice and by writing, the system is equipped with a live chat in which everyone can write and seeing the live surgery was a great formative moment. Each stage of the surgery was visualized on the screen, the relationships with nearby anatomical structures were identified and shown, the movements performed were explained and all the crucial steps were discussed, making surgery easier and safer. During robotic liver resection, the presence of the lesion was verified using an ultrasound robotic probe and the margin to be sectioned was agreed with the surgeons present in telementoring. The quality of remote surgical signals was very good and immediate without latency times useful to explaining the management of intraoperative adverse events.

Throughout the workshop, the participants had the opportunity to dialogue, discuss and interface with expert surgeons from different parts of the world. During all sessions, participants were very satisfied for the good quality of audio and video, for the interactive comparison, for the high visualization and image analysis.

## Discussion

The Metaverse is an extensively studied and tested concept and major scientific advances in the areas of image recording/interpretation are still needed to highlight its real benefit, but the use of the surgical Metaverse has future potential that can be harnessed by combining knowledge and skills acquired from different surgical disciplines.

The Metaverse has great potential for robotic surgeons by guaranteeing a standardized training that goes beyond the possibilities of the single university-hospital, this will grow surgeons who are able to obtain the best quality of their surgery.

Using robotic software for robotic surgery instead laparoscopy with personal computer, the metaverse takes up the old concept of remote surgery by more than one surgeon for telementoring, practical tutoring and emergency surgery directly on the battlefield or on nearest hospital. In fact, if the real hospitals are equipped with robotic surgery and are connected to metaverse, is possible to share the commands to perform the surgical steps. With the Metaverse and VR, today it is possible to transfer all this information and images onto the robotic or laparoscopic monitor overlapping the images and thus acting as a satellite navigator, helping the surgeon in the different steps and increasing the safety and the outcome of the same operation. It is also possible to do discussion, tutoring and distance teaching. The medical responsibilities of remote surgery could be guaranteed by an agreement between the two hospitals or by the same expert surgeons on site as is the case in daily clinical practice. Thinking of having to rely on a simple VR low-cost application might seem unsafe. However, the reliability of new technologies guarantees the function for which they were invented without interference, so VR and Metaverse are certainly suitable for verbal telementoring, remote discussion of clinical cases, image sharing and analysis. The development and the implementation of practical telementoring will be interesting also, because in order to be able to control remotely a surgical robot, it will be necessary to improve the connection between the two consoles in terms of response to movements in order to perform the surgery safely.

Compared to conventional meeting platforms or applications, the Metaverse represents a close space without distractions in which multiple users, personifying with the avatar, can interact together. In the same virtual hospital or operating room, they can share surgery, clinical and scientific data, 3D reconstruction for peri-operative planning or follow up and consultations improving telementoring, telemedicine with second opinion, discussion of clinical cases, surgical practice and images as in an in-person multidisciplinary team.

Furthermore, with the robotic console, we intervene even at kilometers thus reducing costs for a surgical intervention that wants to be limited cost, from the Metaverse to real life.

Finally, the use of this new technology, can certainly facilitate and improve students’ training and education with the possibility to study topics independently, to do simulation with the help of a tutor, consulting the content of VR and sharing video, images or conferences.

## Supplementary Information

Below is the link to the electronic supplementary material.

**Video 1 **Telementoring (MP4 6406 KB)

**Video 2 **Telementoring (MP4 15375 KB)

**Video 3 **Scientific presentation of Metaverse (MP4 5553 KB)

## Data Availability

All data generated or analyzed during this study are included in this published article and its information files.
